# Priority index: database of genetic targets in immune-mediated disease

**DOI:** 10.1093/nar/gkab994

**Published:** 2021-11-09

**Authors:** Hai Fang, Julian C Knight

**Affiliations:** Shanghai Institute of Hematology, State Key Laboratory of Medical Genomics, National Research Centre for Translational Medicine at Shanghai, Ruijin Hospital affiliated to Shanghai Jiao Tong University School of Medicine, Shanghai 200025, China; Wellcome Centre for Human Genetics, University of Oxford, Oxford OX3 7BN, UK

## Abstract

We describe a comprehensive and unique database ‘Priority index’ (Pi; http://pi.well.ox.ac.uk) of prioritized genes encoding potential therapeutic targets that encompasses all major immune-mediated diseases. We provide targets at the gene level, each receiving a 5-star rating supported by: genomic evidence arising from disease genome-wide associations and functional immunogenomics, annotation evidence using ontologies restricted to genes with genomic evidence, and network evidence from protein interactions. Target genes often act together in related molecular pathways. The underlying Pi approach is unique in identifying a network of highly rated genes that mediate pathway crosstalk. In the Pi website, disease-centric pages are specially designed to enable the users to browse a complete list of prioritized genes and also a manageable list of nodal genes at the pathway crosstalk level; both switchable by clicks. Moreover, target genes are cross-referenced and supported using additional information, particularly regarding tractability, including druggable pockets viewed in 3D within protein structures. Target genes highly rated across diseases suggest drug repurposing opportunity, while genes in a particular disease reveal disease-specific targeting potential. To facilitate the ease of such utility, cross-disease comparisons involving multiple diseases are also supported. This facility, together with the faceted search, enhances integrative mining of the Pi resource to accelerate early-stage therapeutic target identification and validation leveraging human genetics.

## INTRODUCTION

Early-stage identification and assessment of genetically validated therapeutic targets can increase the chance of late-stage drug approval. This is extremely important considering two facts. The first fact is that the development process of drug discovery is costly, with an average of ∼$1.4 billion spent per approved drug (1). The second is that the drug attrition rate is extremely high during the drug development process; it is estimated that ∼90% drugs entering phase 1 clinical trials fail to reach approval (2), which is largely explained by a lack of efficacy. Genetic evidence arising from human disease genomics, particularly genome-wide association studies (GWAS), can inform the discovery of therapeutic targets (3,4). Priority index (Pi), made available at http://pi.well.ox.ac.uk, is a comprehensive resource for genetic targets in all major immune-mediated diseases, generated via a well-established genetics-led prioritization strategy. Our approach, Pi (5), leverages the informativeness of GWAS in disease, functional immunogenomics, ontology annotations and network evidence to enhance the drug target prioritization and identification. The Pi approach is purely genetics-driven; we call the prioritization without using any prior existing drug target knowledge as the *discovery* mode. We also prioritize targets in the *supervised* mode that enables the prioritization guided by existing therapeutics in disease. Unless stated explicitly, we are talking about the discovery mode when referring to the Pi approach and resource hereinafter.

Drug targets with genetic support, particularly genetic associations with disease, are twice as likely to be approved as those without support (3). Implementation of genetics-led early target selection, however, remains a prospective area for drug discovery. Linking disease associated loci to the specific genes and pathways responsible for genetic associations is fundamental to drug discovery and poses immense challenges, notably for non-coding loci. By convention, the gene assignment from non-coding loci is based on genomic proximity, and such assignment can be problematic, unavoidably resulting in false negatives given that the effects of loci on gene regulation may be to modulate more distant genes. Functional effects of non-coding loci on genes may involve 3D chromatin structure and are likely to act in a highly cell-type-specific manner. It is increasingly recognized that the assignment of target genes from non-coding loci requires supports from a wide range of cell-type-specific functional genomic datasets, including but not limited to long-range physical chromatin interactions (6) and genetic regulation of gene expression (7). In this aspect, Pi has advanced the progress of this field, both methodologically (5,8) and conceptually (9,10). Here we describe the Pi database contents and the web-based utilities. Our approach with Pi already supports the specific applications (11–15) and is particularly powerful in prioritizing immunomodulatory targets, taking advantages of a large body of immunogenomic datasets that have been generated in a wide variety of immune cell types and states. Moreover, the Pi approach respects the omnigenic model of disease genetic architecture (16), considering potential targets that include not only seed (core) genes directly linked from GWAS summary data and functional immunogenomic data but also non-seed networked (peripheral) genes that are linked to core genes through the knowledge of protein interactions (10). Very often target genes act together in closely related molecular pathways, and current clinical interests targeting pathways highlight the importance of pathway-centric target prioritization and selection. The endpoint (and the uniqueness) of the Pi target prioritization is the identification of a network of highly rated and interconnecting nodal genes that mediate crosstalk between molecular pathways.

Since the Pi approach publication (5), we have improved the Pi resource, including but not limited to: identification of pathway crosstalk genes for each of immune diseases based on the latest KEGG pathway collections, a wide range of annotations on tractability, and druggable pockets predicted using expanded known protein structures. The validity of the resource has been assessed for all diseases where performance evaluation is possible, with improved performance demonstrated over the *status quo*. The most significant progress has been made to enhance the presentation and functionality of the Pi website (and associated database), offering powerful ways to search and use the resource, particularly cross-disease comparisons (not available in our previous publication and elsewhere) that are essential for integrative mining and drug repurposing. To reflect significant improvements and new facilities, in the remaining sections below we first describe the database contents in detail, along with introducing how the resource is generated and how well it performs based on benchmarking. Then, we provide an overview of various utilities available via the website that may interest the users. Finally, we conclude with directions for future developments.

## DATABASE CONTENTS

### Approach summary generating targets at the gene and pathway crosstalk level

**Figure 1.**
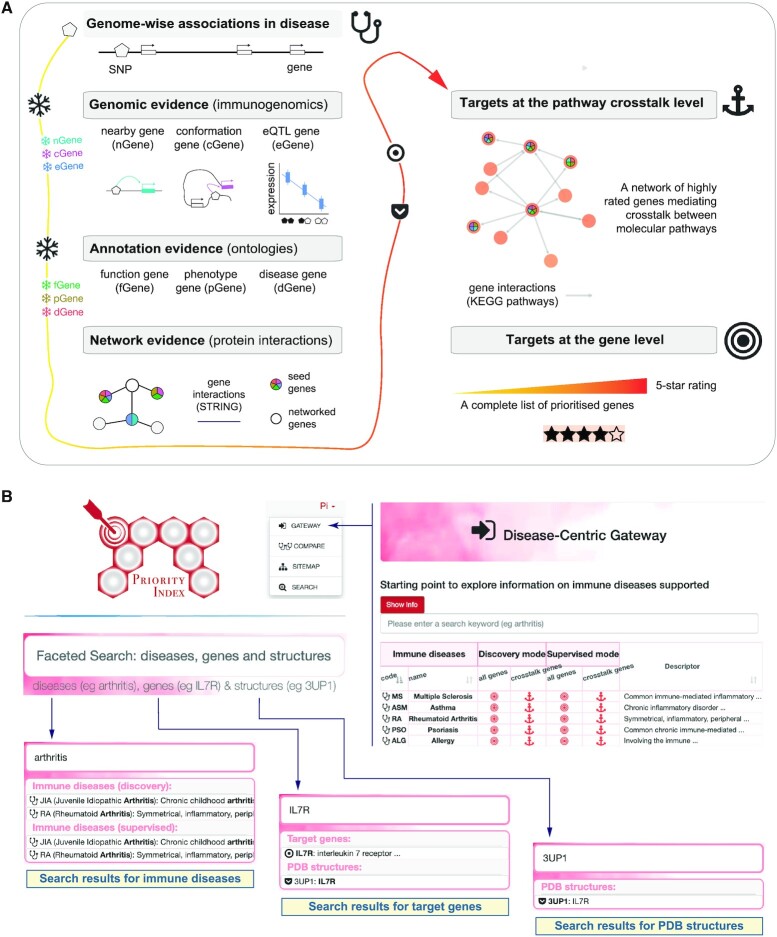
Schematic overview of generating and accessing the Pi resource. (**A**) The workflow of generating the resource, with key steps and concepts illustrated. (**B**) The interface for accessing resource, including the gateway to browser the resource and the faceted search to mine the resource. Notably, the artwork ‘Π’ of the same name is designed to resemble the Pi resource, with glowing circles (symbolising the pill) and red hexagons (the blood) to collectively illustrate the commitment to accelerate genetics-led drug target discovery in immune-mediated disease.

To aid in the users fully understanding the content of the Pi database (Table 1), it is necessary to describe how it is built (Figure 1A). Following a genetics-led viewpoint, we have developed a generic approach that enables the establishment of linking genetic loci to modulated genes and further down to drug targets. The resource has the focus on immune-mediated diseases; all mapped onto Experimental Factor Ontology terms (17) and complemented with expert-level descriptors. In principle the application can be generalized into other disease areas. For detail on the concept, implementation and generalization of translating genetic findings (largely arising from immunogenomic datasets) into drug targets, the users are referred to the previous publications of the approach (5) and an invited review (10). In brief, disease GWAS summary statistics [primarily sourced from GWAS Catalog (18)] is first used to define seed (core) genes under genetic influence, including nearby genes (*nGene*) based on genomic proximity and organization, conformation genes (*cGene*) using promoter capture Hi-C datasets, and expression-associated genes (*eGene*) integrating eQTL datasets. Restricted to seed genes with genomic evidence, ontologies are next used to annotate function genes (*fGene*), phenotype genes (*pGene*) and disease genes (*dGene*). Non-seed networked (peripheral) genes are further identified by exploiting the knowledge of high-confidence gene/protein interactions from the STRING database (19). As a result, a gene-predictor matrix is constructed, containing affinity scores inside. The matrix is used for a genetics-led network-based prioritization. In brief, affinity scores for each predictor are first converted into *P*-like values, and then, for each gene these *P*-values are combined using a Fisher’s combined method, and finally, the combined *P-*values are rescaled into the 0–5 range (i.e. 5-star rating). In doing so, per disease a ranked list of >15 000 targets at the gene level is generated, each gene assigned with 5-star rating and labeled with evidence (i.e. genomic, annotation and network). The Pi approach is unique in identifying a network of highly rated genes that mediate crosstalk between pathways. The identification of this pathway crosstalk is achieved by searching for a subnet of gene interactions [defined by KEGG pathways (20)] enriched with highly rated genes that are linked together through a few less rated genes as linkers. The search is an iterative procedure, ensuring that a desired number (usually 30–50) of genes is identified. This explicit control over a manageable number of genes in the crosstalk is particularly useful for decision-making on which targets are next taken forward for, for example, validation or repurposing. In summary, using the well-established approach applied to the latest data available to us, the Pi database provides the users with two versions of targets: not only a complete list of prioritized genes but also a manageable list of target genes at the pathway crosstalk level (see Table 1).

**Table 1.** A summary of the Pi database contents (the discovery mode; on 15 August, 2021).

		Genomic predictor				Annotation predictor				Benchmarking (AUC)					
Code	Number of targets^a^	nGene	cGene	eGene	Number of seed (core) genes^b^	dGene	pGene	fGene	Number of crosstalk genes^c^	Pi	Naïve^d^	GA^e^	EX^f^	TM^g^	Name
AA	15 195	33	42	174	229	30	16	19	43	-	-	-	-	-	Alopecia areata
AAV	15 167	15	29	10	47	12	7	7	44	-	-	-	-	-	ANCA-associated vasculitis
ALG	15 194	62	112	49	168	34	10	20	36	**0.882**	0.714	0.574	-	0.853	Allergy
AS	15 409	410	682	85	1035	173	72	93	34	**0.818**	0.779	0.598	0.500	0.782	Ankylosing spondylitis
ASM	15 246	125	206	96	359	66	24	41	46	**0.918**	0.760	0.591	0.523	0.839	Asthma
ATD	15 292	134	186	243	458	82	31	47	40	-	-	-	-	-	Autoimmune thyroid disease
BD	15 174	21	55	20	83	27	7	17	47	-	-	-	-	-	Behcet’s disease
CEL	15 286	150	280	177	521	106	43	55	35	-	-	-	-	-	Celiac disease
CRO	15 512	548	994	276	1482	230	96	108	35	**0.920**	0.628	0.537	0.539	0.795	Crohn’s disease
GD	15 217	57	106	58	175	38	15	27	37	-	-	-	-	-	Graves’ disease
Gt	15 173	42	36	24	83	7	4	4	41	**0.794**	0.741	0.586	-	0.766	Gout
JIA	15 235	91	178	81	306	67	30	30	36	0.910	0.747	0.550	0.724	**0.972**	Juvenile idiopathic arthritis
MS	15 360	263	566	187	830	111	63	58	35	**0.891**	0.699	0.517	0.509	0.761	Multiple sclerosis
OA	15 165	19	33	3	45	2	2	3	36	0.718	0.666	0.506	0.570	**0.806**	Osteoarthritis
PBC	15 264	134	229	153	432	74	36	38	36	0.558	**0.667**	0.527	-	0.570	Primary biliary cholangitis
PSO	15 511	588	901	132	1379	220	91	118	33	**0.916**	0.651	0.584	0.531	0.773	Psoriasis
RA	15 432	214	392	571	1014	154	66	70	33	**0.913**	0.688	0.603	0.578	0.848	Rheumatoid arthritis
SAR	15 310	221	177	90	338	45	20	37	40	**0.875**	0.681	0.545	0.510	0.869	Sarcoidosis
SLE	15 397	318	592	174	902	140	50	68	40	**0.939**	0.629	0.611	0.521	0.916	Systemic lupus erythematosus
SSC	15 189	39	81	64	143	32	17	20	36	0.783	0.699	0.535	-	**0.954**	Systemic scleroderma
T1D	15 364	246	368	292	761	117	53	70	50	0.818	0.692	0.537	0.504	**0.866**	Type I Diabetes
UC	15 508	501	885	246	1365	208	92	106	33	**0.914**	0.741	0.567	0.619	0.801	Ulcerative colitis
IGE	15 164	23	38	18	63	20	7	15	36	-	-	-	-	-	IgE and allergic sensitization
IIM	15 220	80	78	37	144	34	8	23	33	-	-	-	-	-	Idiopathic inflammatory myopathies
KD	15 169	27	44	26	79	21	6	8	34	-	-	-	-	-	Kawasaki disease
MG	15 179	16	34	21	63	23	3	15	35	-	-	-	-	-	Myasthenia gravis
NAR	15 173	29	27	9	60	13	8	10	36	-	-	-	-	-	Narcolepsy
PSC	15 422	433	705	340	1122	196	78	111	33	-	-	-	-	-	Primary sclerosing cholangitis
SJO	15 199	44	64	38	119	28	10	14	33	-	-	-	-	-	Sjogren’s syndrome
VIT	15 361	171	227	470	772	99	52	54	34	-	-	-	-	-	Vitiligo

^a^The total number of target genes prioritized.

^b^The total number of genomic seed genes.

^c^The total number of pathway crosstalk genes.

^d^An approach prioritizing a gene by how often it has been targeted by existing approved drugs.

^e^Prioritization based on individual evidence from **G**enetic **A**ssociations (Open Targets).

^f^Prioritization based on individual evidence from gene **EX**pression (Open Targets).

^g^Prioritization based on individual evidence from **T**ext **M**ining (Open Targets).

### Approach novelty and performance

In terms of novelty, the Pi approach can exploit the drug target discovery evidence in the context of their molecular interactions, that is, leveraging the knowledge of protein interactions to identify functionally linked novel targets with no direct genetic evidence [lacking such support in Open Targets (21)]. In terms of performance (see Table 1), benchmarking supports the Pi approach to be highly competitive compared to other genetics-based methods (including Open Targets) and Naïve prediction (the use of the repurposing strategy to prioritize a gene by how often it is targeted by existing drugs). Performance is measured by area under the ROC curve (AUC) separating clinical proof-of-concept targets (reaching development phase 2 and above) from simulated negative controls; details on simulation of negative controls are the same as previously described in (5). The Pi approach is purely genetics-driven, not using the information such as from text mining or gene expression but rather only using primary data (GWAS, and functional genomics in diverse cell types and activation states). Open Targets, also using genetics and genomics for target identification and prioritization, represents the state-of-the-art prioritization. In particular, the Open Targets Genetics Portal prioritizes targets based on GWAS and functional genomics (22), the most relevant approach and resource to Pi. Notably, the overall score from Open Targets already integrates knowledge of approved drug targets, thus excluded for performance evaluation. Instead, used for evaluation in this benchmarking are prioritizations based on individual evidence, including genetic associations (GA; evident from the Open Targets Genetics Portal), gene expression (EX) and text mining (TM). Such benchmarking shows that Pi outperforms the GA and EX prioritizations of Open Targets in all diseases analyzed, and Naïve prediction, which performs better than either GA or EX (Table 1). It is worth noting that Naïve prediction is based on the concept of drug repurposing, thus limited in that it is unable to predict new targets. As compared to the TM prioritization of Open Targets, the Pi performs better in most diseases. Taken together, benchmarking results based on the latest prioritizations support the validity of the Pi resource. There will, however, be a small number of diseases with limited or no genetic data precluding such an approach.

### Target tractability and druggability

Genetic evidence is only one component of the available toolkit for target selection and validation. For each target gene, the Pi database provides information on tractability and druggability. The target tractability is provided based on three drug modalities (21,23,24), including small molecule tractability (buckets 1–8), antibody tractability (buckets 1–9), and PROTAC tractability (buckets 1–8). Also provided is the druggable information, including ChEMBL therapeutic drugs (25), DGIdb druggable gene categories (26), and drug-like binding pockets that are predicted using all known protein structures from the Protein Data Bank (PDB) database (27,28).

## DATABASE WEBSITE

### Data access

The underlying data summarized in Table 1 are available for browsing and download on the Pi website, developed using the next-generation Perl web framework ‘Mojolicious’ and the mobile-first responsive web framework ‘Bootstrap’. The ‘GATEWAY’ navigation tab (Figure 1B) provides the starting point to access genetic targets prioritized in immune-mediated diseases. For each disease, the complete ranked list of target genes and the manageable list of pathway crosstalk genes are provided separately for the discovery mode (i.e. prioritization without using any prior existing drug target knowledge) and the supervised mode (i.e. prioritization through machine learning algorithm ‘random forest’ integrating predictors guided by known drug target knowledge, that is, clinical proof-of-concept targets). Notably, the discovery mode is highly recommended for most users, particularly for those looking for under-explored target candidates, while the supervised mode is suitable for the users seeking to benefit from knowledge of efficacious drugs. In addition to editable files in respective disease-specific pages, the users can download the MySQL relational database along with detailed documentation on table schema and usage. All downloadable files are free for use without any restrictions.

### Faceted search

The faceted search on the Pi website (Figure 1B) is a mining hub, with hyperlinks from the search results. Full text query is supported for: immune diseases, target genes and their known PDB structures. Search results for diseases are linked to disease-specific pages with the tabular display for prioritized target genes. In this display, each gene is assigned with a 5-star rating (and intuitively illustrated), along with an overview of genomic and annotation evidence, the available tractable and druggable information, and estimates of genetic effects on disease. Also supported is the switch, upon clicks, between the discovery and supervised modes, and also between all prioritized genes and genes only in pathway crosstalk.

Search results for a particular target gene are linked to the gene-specific page (generic and irrespective of diseases), showing (i) target general information including external links to a closely related target prioritisation resource [Open Targets (21)], and structural resources for known structures [PDBe-KB (28)] and predicted structures [AlphaFold (29,30)]; (ii) target tractability assessed for three potential drug modalities (i.e. antibody, small molecule and PROTAC) (21,23,24); (iii) target druggable information including DGIdb druggable gene categories (26) and PDB druggable pockets (linked to 3D view of the PDB protein structure embedded with druggable pockets) (27); (iv) target priority, with a tabular illustration of prioritisation for this specific gene in both modes and across diseases (the link also provided, allowing the users to explore the disease-specific page on this specific gene), and drug development phases for respective diseases; and (v) where available, target therapeutics based on information on current therapeutics (including drugs, development phases, target genes, disease indications and primary sources) in the ChEMBL database (25).

For lookups returning a specific PDB structure, the users are provided with opportunities to interactively explore druggable pockets in a 3D view. Gene symbol or access code lookup is supported for all PDB structures. The Pi website is integrated with known protein structures that are predicted to contain drug-like binding sites (i.e. druggable pockets) using the fpocket software (5,31). A gene is defined to be tractable if its known protein structure(s) are predicted to contain druggable pockets. Within the Pi database, druggable pockets for all tractable genes in the human genome are pre-computed and stored as both PDB- and PML-formatted files. These files are available for download, and via NGL Viewer (32), also used for web-based 3D view as cartoon (secondary structure abstraction), color-coded by PDB chains and embedded with druggable pockets. Thus, the support of pocket predictions and 3D view adds an extra dimension to the Pi resource utility. In conclusion, the faceted search is designed for multi-tasking; it does not just provide search results but is also intended to interconnect all database contents and thus enhance cross-referencing utility of the Pi resource.

### Exploring targets at the pathway crosstalk level

**Figure 2.**
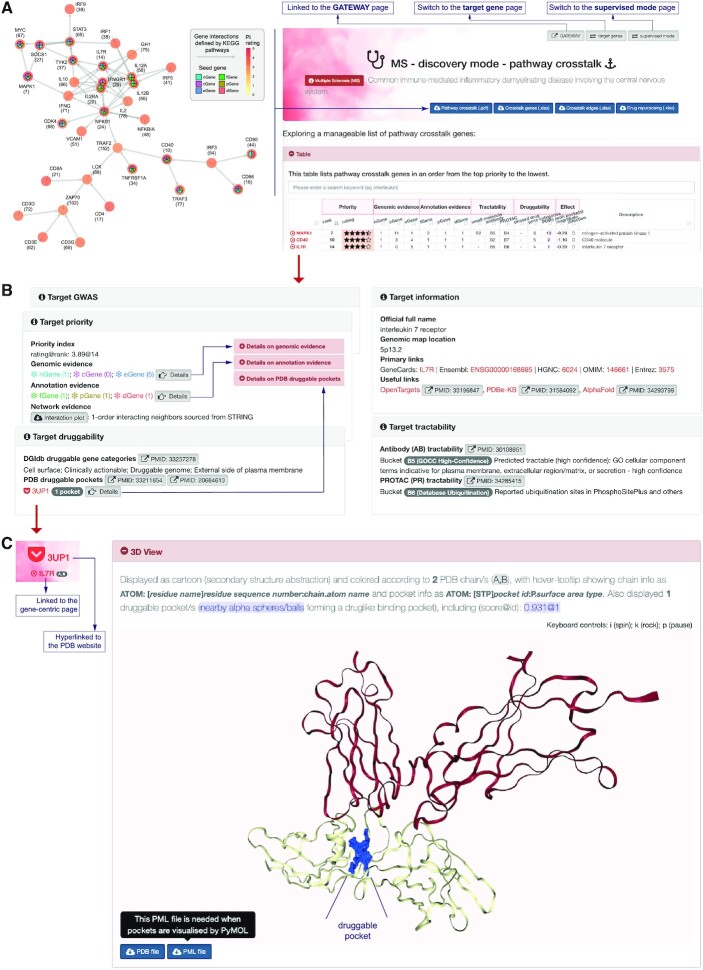
The disease-specific user interfaces for exploring targets at the pathway crosstalk level and associated data, illustrated for multiple sclerosis (MS). (**A**) The page for exploring pathway crosstalk genes. Bottom-right: a tabular display of the top 3 genes in pathway crosstalk together with an overview of evidence, tractability, druggability and effect estimates. Left: network visualization of crosstalk genes, labeled by symbols (rank), colored by rating and embedded with evidence. Also supported upon clicks is instantly switching to, for example, the page for exploring all target genes. (**B**) The page for the rich cross-referencing information on one target gene, *IL7R*. In addition to the general information, the information on priority (and evidence used in Pi for this gene), druggability and tractability are also provided, together with links externally (e.g. AlphaFold) and internally (e.g. details on PDB druggable pockets). (**C**) 3D view of the protein structure, 3UP1. Shown in blue is the druggable pocket. The source files used for viewing are downloadable.

A particular feature of the Pi resource is the ability to provide a manageable list of highly rated nodal genes that mediate crosstalk between molecular pathways. Here we take multiple sclerosis (MS) as an exemplar, a common immune-mediated inflammatory demyelinating disease involving the central nervous system. In Figure 2 with this example we illustrate how the users can access pathway crosstalk genes and associated evidence, which facilitates the target discovery. A total of 35 crosstalk genes are identified, with a tabular summary of these genes and associated evidence, tractability, druggability and effect estimates (Figure 2A). Details on priority (and evidence used), tractability and druggability are provided in the linked gene page, for example, for the gene *IL7R* (Figure 2B). This gene is highly rated (ranked 14th), supported by genomic evidence (*nGene* and *eGene*) and annotation evidence (*fGene*, *pGene* and *dGene*), has tractability based on antibody and PROTAC drug modalities, is annotated by DGIdb druggable gene categories, and contains PDB druggable pockets based on the know protein structure ‘3UP1’ that can be interactively viewed in 3D (Figure 2C). The multiple layers of information on individual genes help the decision-making on target selection and validation.

### Comparing two or more diseases

Increasing evidence has revealed a high degree of genetic overlaps among common diseases. Recently, many attempts have been made at cross-disease comparisons, mostly focusing on pleiotropic loci (33,34). Within the Pi website is a tool called ‘COMPARE’ which compares genetic target genes between any two or more diseases. Upon request, the multi-disease rating score (MRS; ranged from 0 to 1) is calculated to quantify the degree to which a target gene is highly rated across diseases. For the priority being measured in the rank metric, MRS considers the total number (*N*) of diseases under consideration, the number (*nTop*) of diseases in which the target is ranked in the top 1% (the top 150) of the prioritized list for that disease, and the mean rank (*mRank*) of the target only for those *nTop* diseases. The higher values of MRS indicate the more diseases in which a target is highly rated. For multiple diseases in query, COMPARE will identify the list of target genes, ranked by MRS.

**Figure 3.**
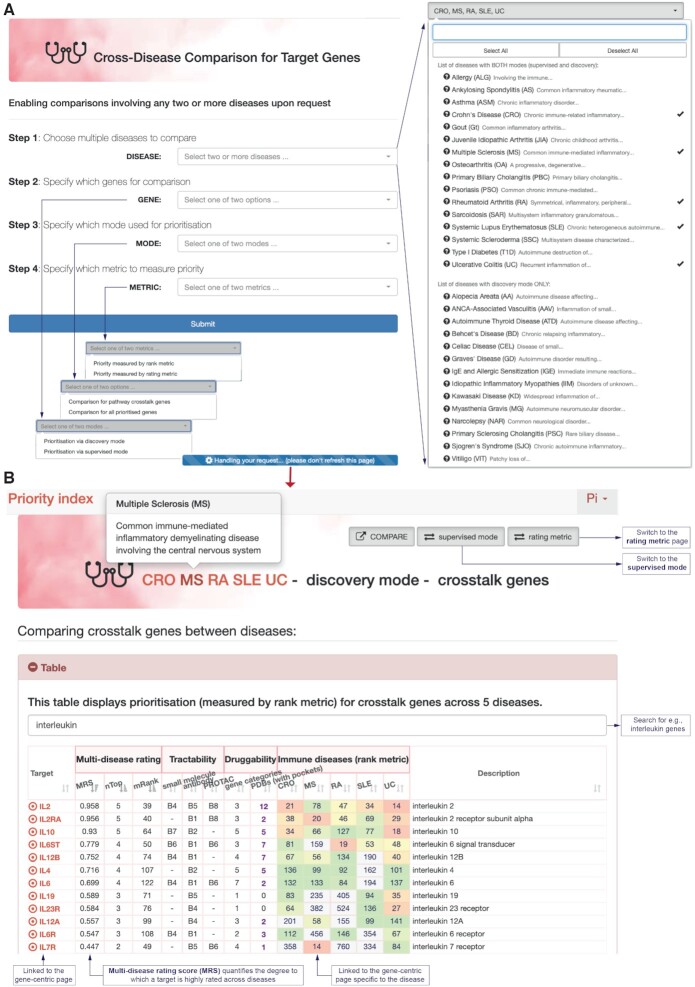
Enabling target gene comparisons involving two or more diseases with the ‘Pi COMPARE’. (**A**) A user request interface allows the selections of immune diseases, target genes, prioritization modes and priority metrics for comparisons. (**B**) The result page provides a summary of target genes in selected diseases, ranked by multi-disease rating score. In addition to the switches, for example, between priority metrics, the users can also explore disease-specific pages, and gene-centric pages (either generic or specific to the disease).

As a proof of principle, we compare five diseases including two autoinflammatory diseases (Crohn’s disease and ulcerative colitis) and three autoimmune diseases (including MS, rheumatoid arthritis and systemic lupus erythematosus) (Figure 3). Selecting these diseases for comparisons can be easily done in a user request interface, together with the choice for the prioritization mode and other options (Figure 3A). Available diseases are organized by the prioritization mode, and in addition to one-by-one selection, selecting all (and deselecting all) is also supported. The comparison results are summarised in a tabular display (Figure 3B), where target genes are ranked by MRS, annotated by tractable and druggable information, and labelled with disease-specific ranks (also color-coded in background). This summary provides a useful means to identify shared target genes, such as *IL2* and the receptor *IL2RA* (35) that are highly rated across diseases, and also to identify genes that are highly rated in a particular disease, for example, *IL7R* in MS (36). Based on shared target genes, the user can explore repurposing opportunity via a heatmap-like illustration and links to gene-centric pages (either generic or specific to the disease). Disease-specific targeting potential can also be explored, particularly considering the tractable and druggable information summarized in the table and detailed in the links.

## CONCLUSION AND FUTURE DEVELOPMENTS

With the increasing rate of growth of human genetic and genomic datasets, the gap between disease associated loci discovery and translational drug discovery is widening. Computational translational approaches and open-access resources are key to shorten such gap, both realistically and rapidly. Centering on the concept of genetic target prioritization, we and others have enhanced the use of multi-layered genomic datasets in target identification and validation. With the unique database ‘Pi’, focusing on immune-mediated diseases, we provide a complete list of all prioritized genes, and more useful for the most users, a manageable list of nodal genes at the pathway crosstalk level. The latter list, together with rich information on tractability and powerful cross-disease mining facilities in the website, represents a *status quo* point with opportunities to take targets forward for validation and repurposing. Benchmarking results show that our genetic target resource recovers proof-of-concept therapeutic targets with a high level of accuracy, and in most diseases, outperforms predictions based on literature mining. Thus the Pi database, in providing target genes on a genome wide and also at the pathway crosstalk level, makes an important contribution to the body of drug target candidates in individual immune diseases and also in two or more combinations of these diseases.

Going forwards, each year we will deliver not only one major release of the Pi resource with new GWAS and functional genomic datasets available to us, but also minor releases with synchronization to the database updates particularly from STRING (protein interactions), PDB (protein structures), Open Targets (target tractability) and KEGG (pathway collections). For the individual user there is capacity to directly use the open-source package (available at http://bioconductor.org/packages/Pi), and we continue to provide high levels of engagement with end-users. As part of the future development, in the first intention we propose to expand the collection of context-specific functional genomic datasets once publicly available, including the recently available eQTL Catalogue (37). It is necessary to increase confidence in prioritizing target genes where regulatory effects of non-coding genomic loci on specific genes are only seen in particular cell types, tissues or conditions. The second intention is to allow applications across the broadest range of diseases and identifications of potential novel under-explored targets. Expanding the disease focus beyond immune-mediated diseases may require the aggregation of prioritization data at the organ or system level. It is necessary particularly for disease areas where sufficient information could be obtained only at the broader phenotype, enabling prioritization. This is motivated by our evolving understanding of disease genetic architecture, in that Mendelian and complex diseases are less dichotomous than previously thought, with significant sharing of genetically implicated pathways (38). This conceptual advance highlights opportunities of leveraging Mendelian genetics in an integrated manner with GWAS for target discovery and validation. The third intension is to enhance druggability assessment for under-explored target genes. Under-explored targets are mostly lacking crystal structures resolved in experiments. The 3D protein structures, predicted computationally via AlphaFold, make it possible to assess their tractability by further predicting druggable pockets. The last but not the least development in the future will be improving tools for cross-disease comparisons and drug repurposing, in a way that they are more focused toward network-based interactive infrastructures.

## DATA AVAILABILITY

Pi can be accessed at http://pi.well.ox.ac.uk.
